# The Impact of Social Determinants of Health, Health Resources, and Environmental Factors on Infant Mortality Rates in Three Gulf Cooperation Council (GCC) Countries

**DOI:** 10.3390/ejihpe15030026

**Published:** 2025-02-21

**Authors:** Moossa Amur Nasser Al Saidi, Rawaa Abubakr Abuelgassim Eltayib, Anak Agung Bagus Wirayuda, Hana Harib Al Sumri, Moon Fai Chan

**Affiliations:** 1 Department of Family Medicine and Public Health, College of Medicine and Health Sciences, Sultan Qaboos University, Muscat 123, Oman; s70215@student.squ.edu.om (M.A.N.A.S.); s136163@student.squ.edu.om (R.A.A.E.); s128499@student.squ.edu.om (A.A.B.W.); alsumry@squ.edu.om (H.H.A.S.); 2 Faculty of Medicine and Health, Institut Teknologi Sepuluh Nopember, Surabaya 60111, Indonesia

**Keywords:** infant mortality rates, social determinants of health, health resources, environmental, gulf cooperation council countries, GCC

## Abstract

Worldwide, there has been a notable decline in the infant mortality rate (IMR) in the last 20 years. Regionally, the Gulf Cooperation Council (GCC) countries echo the global trends to a certain extent. This study aims to explore the impact of social determinants of health (SDOH), health resources (HRS), and environmental (ENV) factors on the IMR in Bahrain, Qatar, and Kuwait. It is a retrospective time-series study using yearly data from 1990 to 2022. Partial Least Square Structural Equation Model (PLS-SEM) was utilized to construct an exploratory model of the IMR for each country. The results showed that SDOH, HRS, and ENV factors influenced IMRs in three GCC countries. In all three countries’ models, only HRS exerted a direct effect on the IMR (Bahrain: −0.966, 95% CI −0.987 to −0.949; Kuwait: −0.939, 95% CI −0.979 to −0.909; and Qatar: −0.941, 95% CI −0.976 to −0.910). On the other hand, ENV factors and SDOH only influenced the IMR indirectly and negatively. Their beta coefficients ranged from −0.745 to −0.805 for ENV factors and −0.815 to −0.876 for SDOH. This study emphasizes the importance of adopting multi-faceted public health strategies that focus on improving socioeconomic conditions, expanding healthcare resources, and reducing environmental degradation. By adopting these multi-dimensional approaches, Bahrain, Qatar, and Kuwait can continue to progress in reducing IMRs and improving overall public health outcomes.

## 1. Introduction

One of the most significant metrics that can accurately indicate a population’s socioeconomic status and general health is its infant mortality rate (IMR). Worldwide, there has been a notable decline in the IMR in the last 20 years (World Health Organization, 2023). For instance, in regions like the Middle East and North Africa, the IMR decreased from 31.9 to 12.2 per 1000 live births between 1990 and 2019 (Sepanlou et al., 2022). Regionally, the Gulf Cooperation Council (GCC) countries echo global trends to some extent. Despite similar economic conditions, some disparities still exist in certain GCC countries’ efforts to reduce the IMR. In Bahrain, the IMR has steadily decreased in recent years, with the current rate in 2024 being recorded at 5.0 deaths per 1000 live births, a 1.86% decline from 2023 (Macrotrends, 2024). Similarly, Qatar has significantly improved by achieving an IMR of 6.50 per 1000 live births in 2023, which is an improvement over their previously recorded records of 6.7 in 2021 and 7 in 2020 (GlobalData, 2023). Comparatively, Kuwait has documented the highest IMR among the three countries, with 8.6 deaths per 1000 live births in 2023 (UNICEF, 2024). This higher IMR suggests that Kuwait may face more challenges related to socioeconomic conditions, healthcare accessibility, and environmental factors.

The socioeconomic conditions in Bahrain, Qatar, and Kuwait have undergone significant changes and rapid modernization, creating healthcare challenges. This element has had a significant impact on public health, mainly in relation to infant mortality rates. Economic inequality is persistent despite the high GDP growth provided by oil revenue and structural reforms. This inequality affects access to healthcare and essential services, particularly among expatriates (SESRIC, 2023; World Bank, 2025). Rapid modernization has changed urban settings and improved environmental sustainability, but it has also affected health conditions owing to some non-communicable diseases (ElMassah & Hassanein, 2023). The healthcare systems present in these countries are advanced in many ways, but they continue to face challenges from non-communicable diseases and socioeconomic inequalities. This directly affects health outcomes such as IMR (Johnson et al., 2024). Several public health initiatives have been launched by these countries in an attempt to combat these issues and improve the overall health of their populations. For instance, The Qatar National Vision 2030 was launched with a multi-faceted approach to improve the health of Qatar’s population as well as the country’s social, economic, and environmental development (Qatar Government Communication Office, 2024). In Kuwait, the country launched a cooperation strategy with the WHO to aid in achieving their health targets, as set out in ‘*Vision 2035*’ (WHO Regional Office for the Eastern Mediterranean, 2023). Bahrain has also joined these countries, striving to attain sustainability, innovation, and fairness in providing healthcare amenities to their population by 2030. Though considerable gains have been achieved through these initiatives, IMR s are still concerning, particularly in Kuwait, with room for improvement in other countries. Hence, shedding light on areas where more effort is needed would further benefit these projects in better tailoring their strategies and reaching their 2030 and 2035 goals.

### 1.1. Literature Review

The literature review mainly consists of areas like social determinants of health (SDOH), health resources (HRS), and environmental (ENV) factors and their influence on the IMR. The review synthesizes global and regional research to contextualize these factors, providing insights into their role in Bahrain, Qatar, and Kuwait.

#### 1.1.1. The Impact of Social Determinants of Health (SDOH) on Infant Mortality Rates

According to the World Health Organization (WHO), SDOH affect individual health, contribute to health inequities, and lead to unjust and preventable disparities in health between different populations (World Health Organization, 2023). SDOH typically encompass factors affecting individuals throughout their lives, and include the broader systems and structures like economic policies, political systems, and social norms (Marmot, 2005). The SDOH framework is the concept of a social gradient of health. Individuals in lower socioeconomic positions will experience worse health outcomes than those in higher socioeconomic positions. Factors such as education, income level, employment, housing conditions, and access to basic amenities can affect health equity (Marmot, 2005). For example, unemployment, poor working conditions, and inadequate access to essential services like quality healthcare can cause health disparities. Some studies have suggested that the SDOH can account for 30 to 55% of health outcomes. This emphasizes the impact of SDOH on health outcomes (Marmot, 2005). In fact, owing to its importance, the SDOH framework is one of the key cornerstones utilized by the Healthy People 2030 initiative launched by the American Office of Disease Prevention and Health Promotion to achieve their goal of improving the overall health and well-being of their population (The Office of Disease Prevention and Health Promotion, 2024).

This SDOH framework is significant for understanding health inequities in countries with various income-levels. Life and healthy life expectancy have increased globally (World Health Organization, 2023). However, some gaps exist, mainly in low- and high-income countries (Braveman et al., 2011). In 2016, over 15 million premature deaths were caused by non-communicable diseases, and the disparities between the wealthy and poor subgroups for diseases like cancer can be seen worldwide (Ishimwe et al., 2023). The under-five mortality rate is more than 8 times higher in Africa compared to Europe (Marmot, 2005). It ranged from as high as 316 deaths in Sierra Leone to as low as 3, 4, and 5 in Iceland, Finland, and Japan, respectively. Among 16 countries, twelve of which were in Africa, the IMRs experienced an exponential rise within just one year in the last decade. For example, Zimbabwe and Botswana both recorded an extreme rise of IMR by 43% and 52% in 1990, respectively (Braveman et al., 2011; Marmot, 2005).

The connection between SDOH and IMRs in both high- and middle-income nations is made by looking at several elements. These include birth and fertility rates, economic factors, and poverty. Increased birth and fertility rates are often associated with increased IMRs, mainly in socioeconomically challenged groups. Reduced fertility rates in high-income countries are mostly related to better maternal healthcare, leading to a decrease in IMRs (Kumar & Cheng, 2023). Economic conditions like unemployment are frequently linked to high stress and limited access to healthcare services, which in turn, leads to increased IMRs. Stability in the economy can help increase access to healthcare, which is crucial for the health of infants and mothers (Ishimwe et al., 2023). Another major factor that can highly impact health is poverty. In both high- and middle-income countries, poor communities have to face high IMRs as they are restricted from accessing healthcare services, proper nutrition, and education. By overcoming poverty, any specific intervention with policies can lead to a decrease in IMRs (Siah & Lee, 2015). Even if SDOH have a negative impact on IMRs, some high-income-generating countries have decreased their effects. They used healthcare policies and societal policies as a way to bring about positive impacts.

As in the GCC countries, the SDOH contribute to health as well as the growth of the economy. They also help us to analyze the roles of gender and those trends in the economy that are mainly related to women. Other factors like cultural rules, legal aspects, and economic policies can influence people and society. The GCC countries had increased economic growth by more than 7% in 2022 (World Bank, 2024). This growth has been supported by reforms that have helped to improve the investment into and the flexibility of the labor market (Yasmeen et al., 2024). These reforms are significant as they helped increase the labor market and employment for women in Saudi Arabia from 17.4% to 36.0% in 2023 (Al Boinin, 2023). The gender dynamics in GCC countries have influenced the ability of women to engage in entrepreneurial activities and are transformed by the expectations of family and society. These socio-cultural elements play a significant role in terms of opportunities and work experience for women (Al Boinin, 2023). However, youth unemployment remains a problem, and young females face higher unemployment rates than males. Because of social and cultural barriers, it is more than five times more likely that females will be unemployed (Fatima & Elbanna, 2022). This also increases economic disparities (Schwartz et al., 2023). These factors lead to health inequities that can impact the health of infants and increase mortality in the region (Eltayib et al., 2023a).

#### 1.1.2. The Impact of Health Resources (HRS) on Infant Mortality Rates

IMRs will be affected by the access and quality of HRS. Some of the important elements are vaccination coverage, skill development, and the availability of healthcare services (Lee et al., 2022; Simmons et al., 2021). These elements can help reduce IMRs in various regions. Vaccination programs can be considered a significant element in the survival of infants because they help in preventing diseases that can lead to the death of the infant (Eltayib et al., 2023b). In some regions like sub-Saharan Africa, a fee must be paid for immunization services. The literature highlights the fact that making vaccines accessible to all is essential for lowering IMRs (Simmons et al., 2021). For the improved outcome of neonatal health, it is essential to have skilled healthcare professionals present during childbirth (Eltayib et al., 2023b). As maternal healthcare services are important, the quality of the care is also comparably important for reducing neonatal mortality rates. This indicates that improvement in both the accessibility and standard of maternal healthcare is significant for effectively lowering the number of neonatal deaths (Lee et al., 2022). However, despite all these advancements, the global disparities in healthcare quality and socioeconomic conditions can be a significant challenge, which means effort is needed to decrease IMRs (Schwartz et al., 2023).

Bahrain, Qatar, and Kuwait are members of the GCC and have similar cultural and historical backgrounds (Khan et al., 2022). However, there are distinct variations in the healthcare systems because of population size, economic development, and specific healthcare challenges. The healthcare system in Bahrain is a combination of public and private sector provision (Katoue et al., 2022). As the system is generally well established, it faces some challenges related to the efficiency and distribution of resources (Alsabah & Alsabti, 2024). In the meantime, Qatar has shown a strong commitment to developing healthcare infrastructure. This has led to increased access and utilization of healthcare services, significantly increasing the number of annual health checkups. The participation increased by 80% between 2017 and 2019 (Al-Abdulla et al., 2024). In Kuwait, people mainly rely on government-provided healthcare services and face challenges in terms of the efficiency of hospitals. The Ministry of Health has delivered 80% of care in Kuwait, but the country has experienced a decrease in technical efficiency (Alsabah & Alsabti, 2024). Regarding immunization coverage, Kuwait has made significant steps towards improving it, as the percentages of children immunized against diphtheria–pertussis–tetanus (DPT), measles, and hepatitis has risen from 67%, 48%, and 38%—respectively—in the 1980s, to around 99% for all of them in the last decade (World Bank, 2025). Similar trends occurred in both Qatar and Bahrain, with vaccination rates experiencing a jump in the percentage of the population immunized against DPT, measles, and hepatitis from around the 30s to around 99% in recent years (World Bank, 2025). A corpus of the literature has highlighted the importance of maintaining sufficient immunization coverage and its significant effect on IMRs (David, 2018; Mouteyica & Ngepah, 2023; Sanchez et al., 2023). Qatar has made considerable advancements related to the comprehensive healthcare screening initiative (Al-Abdulla et al., 2024). Similarly, Bahrain and Kuwait also operate vaccination programs, but their efforts are less documented (Johnson et al., 2024). The investment of Qatar in the healthcare infrastructure and its focus on preventive measures like health checkups and vaccination programs have provided positive results. However, Bahrain and Kuwait can benefit from further analysis of their healthcare systems to identify areas for improvement, mainly in terms of hospital efficiency and vaccination coverage (Eltayib et al., 2023b).

The Gulf states like Bahrain, Qatar, and Kuwait have a shared goal of achieving universal health coverage (Al-Abdulla et al., 2024; Katoue et al., 2022). However, their services and outcomes are different because of differing levels of efficiency. Kuwait has made a notable improvement toward universal health coverage (UHC) as their public hospitals provide mean efficiency rates of up to 86.58%. This proves their commitment to UHC after facing challenges (Alsabah & Alsabti, 2024). Qatar has also taken steps in terms of its healthcare, and uses services and provides preventive care. This has improved the healthcare outcomes in that country (Al-Abdulla et al., 2024). Bahrain, Qatar, and Kuwait have a shared objective in terms of healthcare: universal health coverage. However, their experiences and approaches differ, so understanding these differences may help healthcare providers develop effective healthcare improvement methods.

#### 1.1.3. The Impact of Environmental (ENV) Factors on Infant Mortality Rates

The ENV factors and IMR are connected. Some factors, such as CO_2_ emissions, pollution, and resource availability, can affect the IMR (Kislyak et al., 2024). Nowadays, as of 2023, Qatar is considered one of the major contributors to CO_2_ emissions from fossil fuels and industry as they produced around 38.8 tons per person (t) in that year alone. Following closely behind Qatar are the remainder of the GCC nations, with Saudi Arabia, United Arab Emirates, and Kuwait producing around 22.1 tons, 21.6 tons, and 20.5 tons, respectively. Comparatively, the major polluters worldwide have made significant efforts to reduce their CO_2_ emissions. For instance, during the same year, the USA, Canada, and China managed to limit their emissions to only around 14.3 tons, 14.0 tons, and 8.4 tons, respectively (Our World in Data, 2024). Limiting pollutants in the air is an important issue as the particulate matter within it is linked to issues in the respiratory and cardiovascular regions of the body, which, in turn, lead to an increased risk of infant deaths (Kreutz et al., 2023). The toxic exposure can also cause genetic risks to infants (Kislyak et al., 2024). Air pollution can also occur through extensive energy usage via medical devices. This can indirectly affect public health by contributing to environmental degradation (Roletto et al., 2024). Environmental factors that shape lifestyle choices, such as dietary habits, have also been shown to impact overall health, which can be related to infant mortality (Touil et al., 2023). Although research emphasizes the detrimental effects of environmental factors on IMRs, it also points out that enhanced environmental regulations and healthcare practices could contribute to better health outcomes and lower IMRs over time.

The GCC region experiences significant environmental healthcare challenges because it mainly relies on oil production. The oil production industry will negatively impact air and water resources’ quality, directly affecting public health metrics, mainly IMRs (Nazarpour et al., 2023). The GCC region is strongly affected by harmful air pollutants like particulate matter and nitrogen oxides, which are connected to various health problems. The increased pollution level in Saudi Arabia has been related to reduced life expectancy (Zaidan et al., 2024). Similarly, in Kuwait, there is an increase in particulate matter concentration of up to10 µg/m^3^, which shows the severe health problems of air pollution in the GCC region (Alahmad et al., 2023). Economic growth has also been driven by the consumption of nonrenewable energy resources in the GCC region, negatively affecting environmental sustainability and public health (ElMassah & Hassanein, 2023). From this, we can understand the importance of improving the monitoring and regulatory system to reduce environmental health risks effectively.

### 1.2. Aim and Objectives of the Study

**Figure 1:**
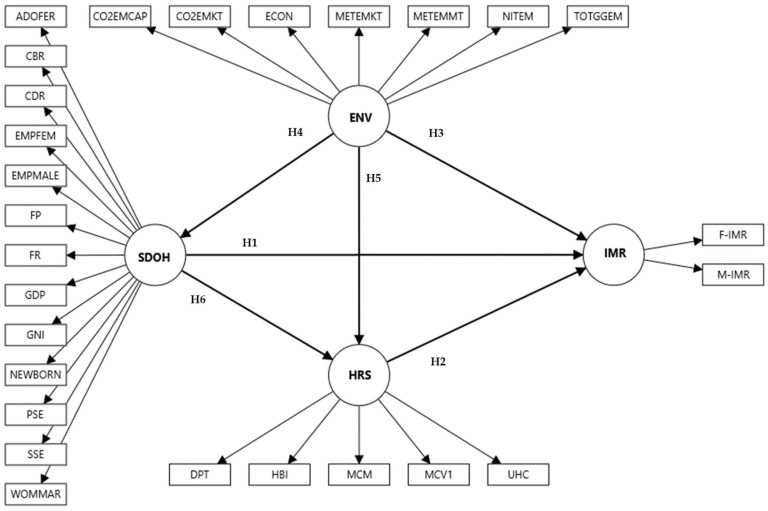
The conceptual model of the impact of SDOH, HRS, and ENV factors on IMR in Bahrain, Kuwait, and Qatar (1990–2022). LV: latent variable; MV: manifest variable; SDOH: sociodemographic on health; ENV: environmental; HRS: health resources; IMR: infant mortality rate; F-IMR: infant mortality rate for females; M-IMR: infant mortality rate for males; UHC: universal health coverage service coverage index; MCV1: newborns vaccinated with MCV1; MCM: method needs for family planning with modern contraception methods; HBI: newborns vaccinated with BCG; DPT: DPT immunization; WOMMAR: percentage of women married; SSE: secondary school education; PSE: primary school education; NEWBORN: gender of a newborn; GNI: gross national income per capita; GDP: GDP per capita; FP: food production index; FR: total fertility rate; EMPFEM: employment to population ratio (females); EMPMALE: employment to population ratio (males); CDR: death rates; CBR: birth rates; ADOFER: adolescent fertility rates; C02EMKT: CO_2_ emissions from oil refineries; CO2EMCAP: CO_2_ emissions per capita; ECON: electricity consumption; METEMKT: methane emissions (kt of CO_2_ equivalent); METEMMT: methane emissions in the energy sector; NITEM: fuel efficiency rate; and TOTGGEM: total greenhouse gas emissions.

This study aimed to understand the direct and indirect impact of the SDOH, HRS, and ENV factors on IMRs in Bahrain, Qatar, and Kuwait. The primary objective is to develop a best-fitting model for each country for IMRs. The second objective is to analyze these factors and compare their impact on IMRs across the three nations by creating an exploratory model using the Partial Least Square Structural Equation Model (PLS-SEM). To achieve the aim of the study, there were six hypotheses to be tested (Figure 1):

**H1:**  *SDOH factors significantly impact IMRs in all three countries.*

**H2:**  *HRS factors significantly impact IMRs in all three countries.*

**H3:**  *ENV factors significantly impact IMRs in all GCC countries.*

**H4:**  *ENV factors significantly impact SDOH factors in all three countries.*

**H5:**  *ENV factors significantly impact HRS factors in all three countries.*

**H6:**  *SDOH factors significantly impact HRS factors in all three countries.*

## 2. Materials and Methods

### 2.1. Research Design and Data Sources

This study used a retrospective time-series design to investigate the impact of SDOH, HRS, and ENV factors on IMR in Bahrain, Qatar, and Kuwait using secondary data from 1990 to 2022. This approach has been used because the study mainly focuses on the measurable relationships between the key variables and IMR. This research focuses on developing models for each country to identify how these determinants can affect IMR. This model can help us understand how each determinant can contribute to IMR variations and provides country-specific models. The data for the independent and dependent variables are sourced from international organizations and country-level health databases to ensure accuracy, consistency, and comparability. Each country’s data were collected in the same timeframe, from 1990 to 2022, for consistent comparison. The data (see Table 1) used for this study are from World Bank (2025), World Health Organization (2024), UNICEF (2024), The Global Economy (2024), Statista (2023), Macrotrends (2024), GlobalData (2023), and SESRIC (2023).

### 2.2. Dependent and Independent Variables

The dependent variable for this study is the IMR, a critical measure of public health. The IMR included the total number of female (F-IMR) and male (M-IMR) infant deaths per 1000 live births within a year. The independent variables have three key categories: SDOH, HRS, and ENV factors. These variables represent a range of socioeconomic, healthcare, and environmental indicators that influence IMR. SDOH are indicated by adolescent fertility rate (ADOFER), birth rate (CBR), death rate in certain year (CDR), employment to population ratio for females (EMPFEM) and males (EMPMALE), percentage of women married in a certain year (WOMMAR), food production index (FP), total fertility rate (FR), gross domestic product (GDP) per capita (GDP), gross national income per capita (GNI), primary school enrolment (PSE), and secondary (SSE) school enrolment. The health resources (HRS) include DPT immunization (DPT), newborns vaccinated with BCG (HBI), family planning with modern contraception (MCM), universal healthcare (UHC) service coverage index (UHC), and newborns vaccinated with MCV1 (MCV1). Environmental (ENV) factors included CO_2_ emissions (CO2EMK), CO_2_ emissions per capita (CO2EMCAP), electricity consumption (ECON), methane emissions (METEMMT) in the energy sector, methane emissions (METRMKT), nitrous oxide emissions (NITEM), and total greenhouse gas emissions (TOTGGEM) (See Table 1).

### 2.3. Statistical Analysis

#### 2.3.1. Analytical Methods

This study employs previous studies that used the PLS-SEM method to assess the complexities between the latent and manifest variables in GCC countries (Eltayib et al., 2023a; Wirayuda et al., 2023). Their predictive nature and ability to establish complex direct and indirect relationships among the variables in small sample sizes make them suitable for this research. Sarstedt et al. (2020) stated that this method uses composite-based modeling that focuses on and predicts outcomes. This research used PLS-SEM to analyze the predictive relationship between SDOH, HRS, and ENV factors in IMRs. Some debates exist about using PLS-SEM in psychological and social sciences (Sarstedt et al., 2020). Although Rönkkö et al. (2016) suggested discontinuing this approach, Rigdon (2016) argued that these misconceptions about applying PLS-SEM overshadow their advantages. PLS-SEM can handle complex and exploratory data using formative models, making it the correct choice for the study. This study mainly tries to explain the variants in IMRs (Kaufmann & Gaeckler, 2015). When using independent variables like SDOH, HRS, and ENV factors, PLS-SEM is mainly designed to accommodate more dependent and independent variables. This model aligns with the needs of the study as the study interrelates the influences of different factors’ impact on IMRs (Eltayib et al., 2023b). This will give modeling flexibility and effectively produce path coefficients (Dijkstra & Henseler, 2015; Shmueli et al., 2016).

#### 2.3.2. Analytical Procedures

The PLS-SEM analysis was conducted using SmartPLS 4.0 (Ringle et al., 2024) software. Various steps are involved in this procedure. First, according to the proposed conceptual model, the modeling specifications involving the model pathway are defined (Gkontelos et al., 2023; Sarstedt et al., 2020). Independent variables like SDOH, HRS, and ENV factors will be specified as latent structures. The dependent variables will be IMRs. Next, path coefficients will be generated, mainly showing the relationship between the independent and dependent variables. The coefficients will help assess their statistical significance (Memon et al., 2021). Various information about factors affecting IMRs in Bahrain, Qatar, and Kuwait will be given in this process. Some key metrics like R^2^, Q^2^, and the SRMR were used for model validation. This model validation is needed to evaluate the quality of the model (Hair et al., 2019). The final step will be applied using the bootstrapping method. In this method, the standard errors and confidence intervals are analyzed to improve the reliability of the loading of each LV on IMRs (Hair et al., 2019). It helps to address the primary objective of this study. Separate models are made for each country, and a comparative analysis is conducted to check how these factors will interact with each other in terms of their impact on IMRs, which helps address this study’s second objective.

#### 2.3.3. Sample and Ethical Considerations

The required samples (number of years) for each country are based on the proposed conceptual model with a set of multiple regressions, including 6 hypotheses. If this study expected a 0.55 effect size, we required at least 32 samples per country (total is 96) to achieve a 5% type I error with 80% power, based on G*Power v3.1 software (Faul et al., 2007). Each country’s data were collected from open sources covering the period from 1990 to 2022 (33 years) (World Bank, 2025; World Health Organization, 2024; UNICEF, 2024; The Global Economy, 2024; Statista, 2023; Macrotrends, 2024; GlobalData, 2023; SESRIC, 2023). This retrospective time-series study uses secondary data with minimal ethical considerations regarding privacy and protection. Ethical approval exemption was obtained from the ethics research committee of the Sultan Qaboos University (MERC#2666). However, care will be taken to ensure the accuracy and consistency of the data across the three countries.

### 2.4. Analysis

Based on the prior systematic review (Eltayib et al., 2023a) and a study in Oman (Eltayib et al., 2023b), the conceptual model is presented in Figure 1. A comprehensive overview of the manifest variables (MVs) and latent variables (LVs) is provided in Table 1. The model incorporates MVs that represent their corresponding LVs. According to the systematic review in GCC countries (Eltayib et al., 2023a), all items significantly impact IMRs. The structural or inner model examines the relationships between the specified LVs, as shown in Figure 1. Additionally, indirect and total effects are automatically available through SmartPLS 4.0 (Ringle et al., 2024). Negative loading MVs in the PLS algorithm are adjusted following PLS-SEM guidelines. The model’s robustness was evaluated using multiple reliability and validity measures. Each MV was confirmed to have statistically significant loadings at the 5% level. Internal consistency reliability for LVs was assessed through Cronbach’s alpha (CA), composite reliability (CR), and Rhô-A, all requiring a minimum threshold of 0.7 (Sarstedt et al., 2020). Convergent validity was verified using the average variance extracted (AVE), with a cut-off of 0.50 (Rönkkö et al., 2016). Discriminant validity between two LVs is confirmed when the upper bound of the confidence interval (CI) for the HTMT criterion is below 1 (Hair et al., 2019). Predictive capability is indicated by R^2^ and Q^2^, with an R^2^ > 0.25 recommended and a Q^2^ > 0.35 considered strong (Sarstedt et al., 2020). The f^2^ also gauges the significance of direct effects, with values >0.35 indicating strong significance (Faul et al., 2007; Sarstedt et al., 2020). Path coefficients were estimated through bootstrapping with 10,000 subsamples, and coefficients greater than an absolute 0.20 were deemed significant (Sarstedt et al., 2020).

## 3. Results

### 3.1. The Bahrain Model

**Figure 2:**
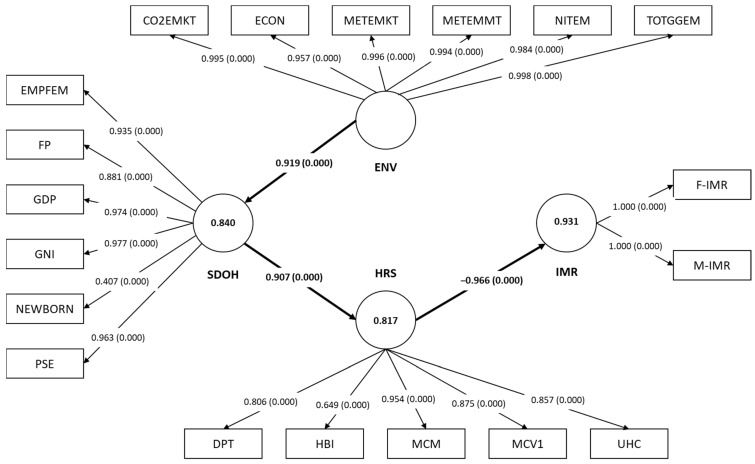
The final model of the impact of SDOH, HRS, and ENV factors on IMRs in Bahrain, (1990–2022). SDOH: sociodemographic on health; ENV: environmental; HRS: health resources; IMR: infant mortality rate; F-IMR: infant mortality rate for females; M-IMR: infant mortality rate for males; UHC: universal health coverage service coverage index; MCV1: newborns vaccinated with MCV1; MCM: method needs for family planning with modern contraception methods; HBI: newborns vaccinated with BCG; DPT: DPT immunization; PSE: primary school education; NEWBORN: gender of a newborn; GNI: gross national income per capita; GDP: GDP per capita; FP: food production index; EMPFEM: employment to population ratio (females); C02EMKT: CO_2_ emissions from oil refineries; ECON: electricity consumption; METEMKT: methane emissions (kt of CO2 equivalent); METEMMT: methane emissions in the energy sector; NITEM: fuel efficiency rate; and TOTGGEM: total greenhouse gas emissions. The circular shapes represent the LVs, while the rectangular shapes represent the MVs. The numbers in the arrows between the LVs and MVs represent the factor loading of each MV, while the numbers between the brackets reflect the loadings’ *p*-values. The numbers inside the LV circle indicate the R^2^ value. The numbers in between the arrows of the pathways between the LVs represent the model’s beta coefficients and their *p*-values are given between the brackets. All the values inside the diagram above are significant at *p* < 0.05.

The final model that best represents the determinants of IMRs in Bahrain is given in Figure 2. Overall, the IMR was reflected by both male and female IMRs (each having a loading of more than 0.7 and significant at *p* < 0.001). For the ENV factors determinant, it was reflected by six MVs in the final model (CO2EMKT, ECON, METEMMT, METEMKT, NITEM, and TOTGGEM), all boasting factor loadings >0.7 and significant at *p* < 0.001. Next, the SDOH LV was reflected by six MVs out of the original proposed seven MVs: EMPFEM, FP, GDP, GNI, NEWBORN, and PSE. Lastly, the HRS LV was reflected by five MVs, all similar to the ones in the hypothetical model: DPT, HBI, MCM, MCV1, and UHC. To be included in the final model, all of these MVs had factor loading values of more than 0.7 and were significant at *p* < 0.05. For a more detailed overview of the LVs, MVs, and their loadings, refer to Table 2.

After ensuring that Bahrain’s final model contained suitable MVs, the reliability, validity, and predictability of each LV had to be evaluated. As such, for the reliability, CA, CR, and Rhô-A measures were utilized. A more in-depth summary of these values is given in Table 3. In Bahrain, the final model was reliable as all reliability measures were above the minimum threshold of 0.7. For the HRS LV, it had a CA of 0.890, a CR of 0.919, and a Rhô-A of 0.927. Similarly, the SDOH (CA = 0.932, CR = 0.951, and Rhô-A = 0.974), ENV factors’ determinants (CA = 0.995, CR = 0.996, and Rhô-A = 0.995) and IMR LV (CA = 1.000, CR = 1.000, and Rhô-A = 1.000) showed appropriate reliability measures. In addition, the final model showed a good convergent validity with an AVE value of 0.696 for the HRS, 0.775 for the SDOH, 0.974 for the ENV factors determinant, and 1.000 for the IMR LV. As for the discriminant validity, the HTMT criterion for each relationship in the final model fell below 1.0, thus establishing discriminant validity. Lastly, the model’s predictability was evaluated via its R^2^ and Q^2^ values. The final model showed that it can explain about 82.3% of the variance in the HRS determinant, 84.5% of the variance in the SDOH LV, and 93.3% of the variance in IMRs. Regarding the Q^2^ values, the final model showed good predictability with Q^2^ values ranging from 0.541 for HRS, 0.837 for SDOH, and 0.764 for IMR (Table 3).

Finally, the Bahrain’s final model relationships and pathways had to be evaluated. A thorough summary of the values is given in Table 4. From the initial proposed six hypotheses, only three hypotheses were validated in the final model (H2, H4, and H6). The second hypothesis is present in the final model, as HRS directly, significantly, and negatively affect IMRs (β = −0.966, CI: −0.987 to −0.949, and *p* < 0.001) (Figure 2). The fourth hypothesis states that the ENV factors determinant in Bahrain exerts a significant direct effect on the SDOH, with a beta coefficient of 0.919 (CI: 0.829–0.993) and a *p* value of <0.001. The last hypothesis to be validated by the final model is the sixth one, which states that the SDOH LV influences the HRS determinant directly (β = 0.907, CI: 0.873–0.941, and *p* < 0.001). All of these three relationships showed strong significance, with f^2^ values ranging from 4.654 to 13.912, far surpassing the 0.35 threshold.

Out of all the three determinants, only the HRS exerted a direct effect on IMRs, while the ENV factors and SDOH determinant only influenced IMRs indirectly, with a total significant effect of −0.805 and −0.876, respectively (Table 4).

### 3.2. The Kuwait Model

**Figure 3:**
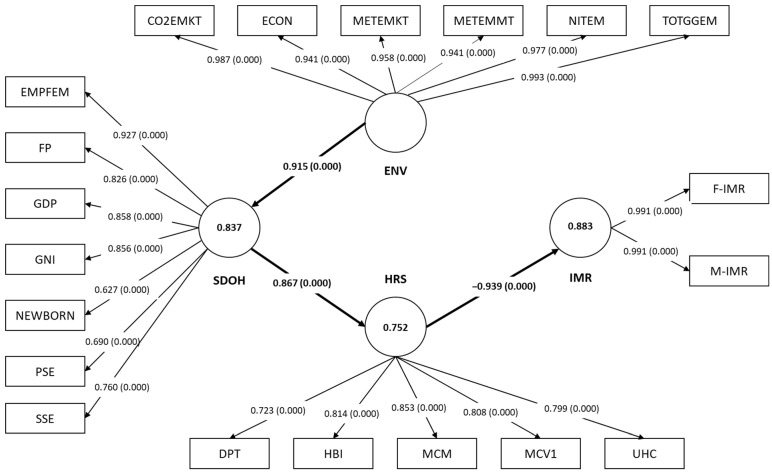
The final model of the impact of SDOH, HRS, and ENV factors on IMRs in Kuwait (1990–2022). SDOH: sociodemographic on health; ENV: environmental; HRS: health resources; IMR: infant mortality rate; F-IMR: infant mortality rate for females; M-IMR: infant mortality rate for males; UHC: universal health coverage service coverage index; MCV1: newborns vaccinated with MCV1; MCM: method needs for family planning with modern contraception methods; HBI: newborns vaccinated with BCG; DPT: DPT immunization; SSE: secondary school enrolment; PSE: primary school education; NEWBORN: gender of a newborn; GNI: gross national income per capita; GDP: GDP per capita; FP: food production index; EMPFEM: employment to population ratio (females); C02EMKT: CO_2_ emissions from oil refineries; ECON: electricity consumption; METEMKT: methane emissions (kt of CO_2_ equivalent); METEMMT: methane emissions in the energy sector; NITEM: fuel efficiency rate; and TOTGGEM: total greenhouse gas emissions. The circular shapes represent the LVs, while the rectangular shapes represent the MVs. The numbers in the arrows between the LVs and MVs represent the factor loading of each MV, while the numbers between the brackets reflect the loadings’ *p*-values. The numbers inside the LV circle indicate the R^2^ value. The numbers in between the arrows of the pathways between the LVs represent the model’s beta coefficients and their *p*-values are given between the brackets. All the values inside the diagram above are significant at *p* < 0.05.

In Kuwait, the final model showed that the IMR LV was best reflected by both male and female IMRs, with factor loadings equal to 0.991 for both MVs and significant at *p* < 0.05. Generally, all of the proposed MVs were found to have sufficient factor loadings and significantly reflect the HRS, SDOH and ENV factors’ determinants in Kuwait’s final model (Table 2). For a graphical representation of the final model for Kuwait, refer to Figure 3.

The final model also showed appropriate reliability measures. HRS metrics were CA = 0.870, CR = 0.899, and Rhô-A = 0.895. Moreover, the SDOH had a CA of 0.902, a CR of 0.924, and a Rhô-A of 0.913, while the ENV factors displayed a CA value of 0.986, a CR value of 0.988, and a Rhô-A value of 0.986. The IMR LV also showed appropriate reliability measures (CA = 0.982, CR = 0.991, and Rhô-A = 0.983) (Table 3). Further, both convergent and discriminant validity were appropriately established in the final model via the AVE and HTMT measures, respectively. The AVE values for all the LVs surpassed the 0.5 threshold (for HRS = 0.641, for SDOH = 0.637, for ENV = 0.934, and for IMR = 0.982). The upper limit of the CI for the HTMT criterion is below one for all LVs, thus ensuring discriminant validity for the final model. Kuwait’s model also showed that it could account for 88.3% of the variation in IMRs, 75.2% of the variation in the HRS, and 83.7% of the variation in the SDOH. Its Q^2^ values surpassed 0.35, thus reflecting the strong predictability of the final model (Table 3).

Regarding the validation of the proposed hypotheses, Kuwait’s final model pathways echoed that of Bahrain’s, as only three hypotheses (H2, H4, and H6) were significant out of the original six hypotheses. The final model reveals that only the HRS exerted a direct effect on IMRs (H2), with a beta coefficient of −0.939 (*p* < 0.001). In addition, the ENV factors’ determinant influenced the SDOH (H4) directly and positively (β = 0.915, CI: 0.855–0.971, and *p* < 0.001). The SDOH determinant also exerted a positive direct effect on the HRS (H6: β = 0.867, CI: 0.831–0.944, and *p* < 0.001). Further, the f^2^ values for these relationships ranged from 3.038 for H6, 5.132 for H4, to 7.518 for H2 (Table 4), indicating strong relationships between the above-mentioned determinants. Regarding the IMR LV, also echoing Bahrain’s model, the SDOH and ENV factors only influenced IMRs indirectly, with a negative total effect of −0.815 (*p* < 0.001), and −0.745 (*p* < 0.001), respectively.

### 3.3. The Qatar Model

**Figure 4:**
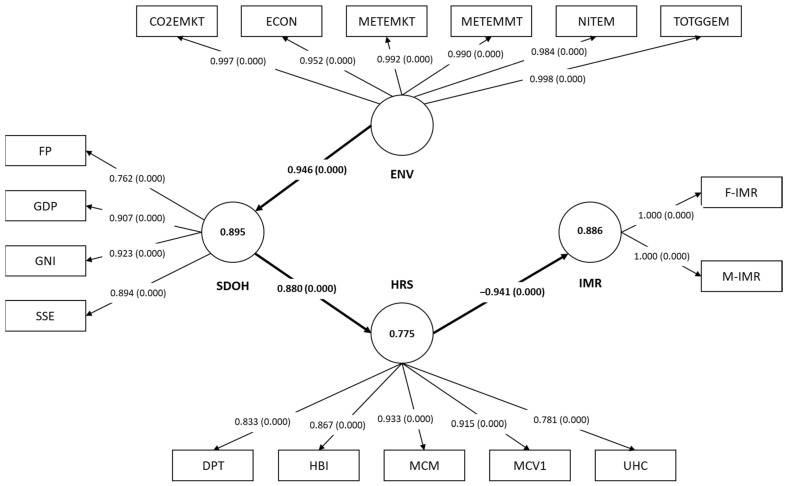
The final model of the impact of SDOH, HRS, and ENV factors on IMRs in Qatar (1990–2022). SDOH: sociodemographic on health; ENV: environmental; HRS: health resources; IMR: infant mortality rates; F-IMR: infant mortality rate for females; M-IMR: infant mortality rate for males; UHC: universal health coverage service coverage index; MCV1: newborns vaccinated with MCV1; MCM: method needs for family planning with modern contraception methods; HBI: newborns vaccinated with BCG; DPT: DPT immunization; SSE: secondary school enrolment; GNI: gross national income per capita; GDP: GDP per capita; FP: food production index; C02EMKT: CO_2_ emissions from oil refineries; ECON: electricity consumption; METEMKT: methane emissions (kt of CO_2_ equivalent); METEMMT; methane emissions in the energy sector; NITEM: fuel efficiency rate; and TOTGGEM: total greenhouse gas emissions. The circular shapes represent the LVs, while the rectangular shapes represent the MVs. The numbers in the arrows between the LVs and MVs represent the factor loading of each MV, while the numbers between the brackets reflect the loadings’ *p*-values. The numbers inside the LV circle indicate the R^2^ value. The numbers in between the arrows of the pathways between the LVs represent the model’s beta coefficients and their *p*-values are given between the brackets. All the values inside the diagram above are significant at *p* < 0.05.

A summary illustration of the Qatar final model is provided in Figure 4. Firstly, regarding which MVs were found to best reflect each LV, IMRs, the HRS, and the ENV factors were best reflected by all of their original proposed MVs (two MVs for the IMRs: F-IMR and M-IMR, five MVs for the HRS: DPT, HBI, MCM, MCV1, and UHC, and six MVs for ENV factors: CO2EMKT, ECON, METEMMT, METEMKT, NITEM, and TOTGGEM). The SDOH determinant was best reflected by only four of its original proposed MVs: FP, GDP, GNI, and SSE (Table 2).

Qatar’s final model displayed good reliability metrics, with the HRS determinant having a CA of 0.918, CR of 0.938, and Rhô-A of 0.935. Similarly, the remaining LVs also showed food reliability values (For SDOH: CA = 0.895, CR = 0.928, and Rhô-A = 0.904. For ENV: CA = 0.994, CR = 0.995, and Rhô-A = 0.994.). For the IMRs, all three metrics displayed values of 1 (Table 3). Furthermore, both convergent and discriminant validity was well established in the final model, with all four LVs displaying AVE values surpassing the 0.5 threshold, and an HTMT criterion falling below 1 for each relationship (Table 3). Moreover, the R^2^ values showed that a good amount of the variability in each LV was explained by the final model (For IMR = 0.886, for HRS = 0.775, and for SDOH = 0.895.). Lastly, the Q2 metrics point towards the final model having good predictability (For IMR = 0.709, for HRS = 0.669, and for SDOH = 0. 889.).

Concerning the significant pathways, only three hypotheses (H2, H4, and H6) were validated in the final model. H2: the HRS exerted a direct, but negative effect on the IMRs (β = −0.941, CI: −0.976 to −0.910, and *p* < 0.001). H4: the ENV factors’ determinant influenced the SDOH directly (β = 0.946, CI: 0.887–0.989, and *p* < 0.001), and H6: the SDOH exerted a direct impact on the HRS determinant (β = 0.880, CI: 0.847–0.932, and *p* < 0.001). All of these pathways were strongly significant relationships as they displayed f^2^ values above 0.35 (Table 4). Lastly, Qatar’s final model showed that while the HRS affected the IMRs directly, the remaining LVs, -SDOH and -ENV, only influenced the IMRs indirectly. For SDOH the total effect was net negative (β = −0.828, *p* < 0.001), and for ENV it was also a negative net effect (β = −784, *p* < 0.001).

## 4. Discussion

Through a retrospective time-series study, this study has examined the influence of SDOH, HRS, and ENV factors on IMRs in Bahrain, Qatar, and Kuwait. The result shows that in all three countries there is a significant direct relationship between HRS and IMRs and an indirect impact between ENV factors and SDOH factors on IMRs.

SDOH encompass factors such as socioeconomic status, education, employment, and fertility rates, all of which play an important role in determining the health outcomes of a population (Braveman & Gottlieb, 2014). This study’s findings suggest that female employment rates can influence infant mortality. This is consistent with the findings of other studies in the GCC region (Eltayib et al., 2023a, 2023b). One explanation is that mothers who cannot take sufficient time off may face challenges in providing direct care, breastfeeding, or attending regular healthcare appointments for their infants, which are critical for reducing infant mortality (Siah & Lee, 2015). The results from this study show that the female employment rate significantly affects the IMR (ElMassah & Hassanein, 2023). In Kuwait and Bahrain, higher female employment rates have been linked with lower IMRs (Schwartz et al., 2023). The reason is that women working in employment promotes better healthcare decision-making during pregnancy and allows them to access healthcare and family planning services (Salam & Al-Khraif, 2020). This directly reduces the risks of malnutrition, infections, and other preventable causes of infant deaths. Our models showed that the FP index has significantly impacted IMRs in all three countries. A previous study has shown that ensuring a steady food supply through a high FP index is crucial for lowering IMRs in the GCC countries (Al-Saidi & Saliba, 2019). Similarly, Al-Saidi and Saliba (2019) reported that the FPI reflects food availability within a country and is directly linked to nutritional outcomes, affecting IMRs in all three countries. A study analyzing the GDP per capita of 193 countries revealed a strong negative relationship with IMRs, indicating that economic growth facilitates better healthcare outcomes and social safety nets for families (Schwartz et al., 2023). Our findings align with the FP index and show that GDP and GNI significantly impact IMRs in all three countries. To ensure a stable food supply in these countries, they must be high-income nations, so a strong economy can enable these countries to allocate significant resources to healthcare, maternal and child health services, and the provision of essential nutrients for infants, all of which contribute to lower IMRs (Salam & Al-Khraif, 2020). This study found that primary and secondary education enrolment are factors impacting IMRs. One reason is that these countries with high educational access have contributed to lower IMRs by increasing awareness of proper prenatal and neonatal care. Educated families are more likely to seek timely medical assistance, understand the importance of immunizations, and maintain hygienic practices that prevent infant disease (ElMassah & Hassanein, 2023; Eltayib et al., 2023b).

The availability of HRS, like immunization coverage, family planning services, and universal health coverage, also plays an important role in reducing infant mortality in these three countries (Katoue et al., 2022). Vaccinations like DPT and MCV1 (measles) are important as they show healthcare access in a country (Salam & Al-Khraif, 2020). In low-income countries, such as those in sub-Saharan Africa, financial constraints often mean that immunization services are not free for all infants, and that requiring fees contributes to higher IMRs (Simmons et al., 2021). In contrast, Bahrain provides free DPT immunization coverage for all infants, which reached 99% in 2022, resulting in a reduction in the IMR to 8.3 deaths per 1000 live births. Similarly, Qatar and Kuwait have increased immunization rates to 98%, leading to a reduction in the IMR of up to 67%. IMRs are also influenced by the implementation of UHC programs in many countries. Previous studies (Preker et al., 2021; Sharma et al., 2023) have reviewed UHC programs across various nations and concluded that UHC is an achievable goal, primarily attainable by high-income countries, often through core government funding. Kuwait and Qatar have established comprehensive healthcare programs that include maternal and child healthcare services (Katoue et al., 2022), contributing to safer pregnancies and births (Sharma et al., 2023). The index of such services in Kuwait was 89 in 2022. Some modern family planning methods have helped women in Saudi Arabia to have a gap between pregnancies (Alenezi & Haridi, 2021). This helps them reduce the risk of pregnancies, reducing the IMR. Modern contraception has increased by 30 percent in 2022 (Mahfouz et al., 2023). This has been related to the overall decrease in high-risk pregnancies and half of the reduction in infant mortalities. Perhaps these three target countries can learn from Saudi Arabia by implementing these methods to reduce the IMR in the future.

Research shows that even air pollution exposure below these standards can increase neonatal mortality risks in countries like India, China, and other Southeast Asian countries. For example, a study in Southern Asia highlighted that over 75% of neonatal deaths occur in regions with hazardous air pollution levels (Anwar et al., 2021). In a few Araba countries like Iran, a 10-year study reported that environmental factors like air pollution, greenhouse gas emissions, and access to clean energy are other determinants of infant mortality (Nazarpour et al., 2023). Oil producers like the GCC countries will face various challenges related to air pollution. This will affect public health, mainly in infants (Khan et al., 2022). Increased carbon dioxide emissions will cause respiratory problems in infants, possibly leading to death (Anwar et al., 2021). In Kuwait, CO_2_ emissions have increased by up to 35 metric tons per capita in 2022. This can be related to the slightly increased IMR of 9.2 compared to other countries (AlRukaibi & AlSalem, 2022). Qatar is also a significant emitter of CO_2_, but they have implemented cleaner energy policies that have helped reduce the impact on the mortality rate. Greenhouse gases like methane and nitrous oxide can be related to premature birth and infant respiratory diseases. 

Bahrain, Kuwait, and Qatar have implemented policies that help them reduce greenhouse gas emissions (AlRukaibi & AlSalem, 2022; Shannak & Contestabile, 2022). This helped them increase fuel efficiency and reduce the flaring during oil production. A 15% reduction has been observed in Kuwait for greenhouse gas production, and there was an 8% decrease in the IMR simultaneously (AlRukaibi & AlSalem, 2022). In addition, Qatar has also invested in solar energy, which can help reduce pollution (Shannak & Contestabile, 2022) and may have resulted in a reduction in the IMR to 62% in 2022. The conversion towards cleaner energy sources has been important in decreasing the IMR in other GCC countries.

Overall, this study’s findings have provided valuable information regarding how these factors interact and influence IMRs across different contexts, revealing details that can guide policy and improve public health outcomes in the GCC region. For example, developing policy interventions can be tailored to address the SDOH, HRS, and ENV factors contributing to reducing the IMR, such as maternal education, income level, and air pollution. In addition, such an understanding of the IMR determinants can help in improving health system financing efficiency through improved dynamic allocation of medical resources across different demographic groups (e.g., low, middle, and high income). Further, in order to aid in achieving their national visions by the year 2030, Bahrain and Kuwait need to further tailor their national strategies by addressing factors such as female employment, more comprehensive childhood vaccinations, and stricter environmental policies. For Qatar, focusing more on factors such as education, immunization coverage, as well as on cleaner energy sources will prove beneficial towards further reducing their IMRs and improving the overall health of their population. In addition, this study’s findings further elucidate the point that adopting a multi-sectoral approach that integrates health, education, economic, and environmental sectors as well as legislative and organizational policies is the most efficient pathway to further reduce the chances of infant deaths. Moreover, this multi-faceted approach will aid exponentially in achieving their already existing initiatives’ targets. 

This study has several strengths. For instance, it is the first of its kind that took into account the comparison of the factors that may affect IMRs in the GCC countries. In addition, this study is the largest study of its kind in terms of the length of the period covered (33 years). Furthermore, the use of structural equation modeling gives a distinct and more in-depth insight into the intricacies of how several factors interact with each other to affect IMRs. Despite these strengths, some limitations in this study are also acknowledged. For example, population size and density variations across countries introduce intra-group heterogeneity and complicating cross-country comparisons in population-based research. Although the studied countries share geographic and economic similarities, they differ significantly in terms of population size, gender proportion, financial structure, cultural practices, and health policies. These differences may limit the generalizability of findings and pooled results require cautious interpretation, highlighting the need for country-specific analyses to understand better the factors influencing IMRs. Additionally, determinants of IMRs may vary between females and males across the studied countries, warranting further research on this issue. While using panel data and PLS-SEM offers valuable insights into complex relationships, it does not establish cause-and-effect links. Moreover, unmeasured confounders, such as lifestyle behaviors and legislative changes, may affect IMRs but were not captured due to data limitations. The dataset, spanning 33 years annually per country, may also increase the risk of type II errors and reduce statistical power.

## 5. Conclusions

This study showed that SDOH, HRS, and ENV factors influenced IMRs in three GCC countries. While SDOH, like high female employment and educational rate in primary and secondary schools, stable GDP, GNI, and FPI contribute to better healthcare access and outcomes that reduce IMRs, the HRS mainly cover immunization coverage and UHC, directly affecting IMRs by enhancing infant health services. Environmental factors like pollution present ongoing challenges, though clean energy initiatives have proven beneficial. GCC countries have made substantial progress in reducing IMRs, but continued focus on improving these determinants will be crucial for further reductions. The study highlights the need for multi-faceted public health strategies addressing socioeconomic, healthcare, and environmental challenges. Targeted policies improving socioeconomic conditions, expanding healthcare access, and reducing environmental degradation can help Bahrain, Qatar, and Kuwait reduce IMRs and enhance public health outcomes. Future research should focus on country-specific determinants of IMRs, offering deeper insights into unique cultural, economic, and environmental factors. Such tailored approaches could lead to more effective, localized interventions, strengthening public health in each country.

## Figures and Tables

**Table 1:** List of Latent and Manifest Variables in the Conceptual Model.

LV	MV (Unit)	Abbreviation
IMR *	Infant Mortality Rate, total female infant death per 1000 live births (year)	F-IMR
	Infant Mortality Rate, total male infant death per 1000 live births (year)	M-IMR
SDOH ^	Adolescent fertility rate in a certain year	ADOFER
	Birth rate in a certain year	CBR
	Death rate in a certain year	CDR
	Employment to population ratio (females) in a certain year	EMPFEM
	Employment to population ratio (males) in a certain year	EMPMALE
	Percentage of women married in a certain year	WOMMAR
	Food Production Index in a certain year	FP
	Total fertility rate in a certain year	FR
	GDP per capita (Local Currency Unit)	GDP
	Gross national income per capita in a certain year	GNI
	Gender of the newborn in a certain year	NEWBORN
	Primary School Enrolment in a certain year	PSE
	Secondary School Enrolment in a certain year	SSE
ENV ~	CO_2_ emission or oil refineries	CO2EMKT
	CO_2_ emission per capita	CO2EMCAP
	Electricity Consumption in a certain year	ECON
	Methane emissions in the energy sector (thousand metric tons of CO_2_ equivalent)	METEMMT
	Methane emissions (kt of CO_2_ equivalent)	METEMKT
	Nitrous oxide emissions (thousand metric tons of CO_2_ equivalent)	NITEM
	Total greenhouse gas emissions (kt of CO_2_ equivalent)	TOTGGEM
HRS #	DPT immunization	DPT
	Newborns are vaccinated with BCG.	HBI
	Method needs for family planning with modern contraception methods	MCM
	Newborns vaccinated with MCV1 in a certain year	MCV1
	Universal health coverage (UHC) service coverage index in a given year.	UHC

LV: latent variable. MV: manifest variable. SDOH: sociodemographic on health; ENV: environmental factors; HRS: health resources; IMR: infant mortality rate; *: sources from World Health Organization (2024), GlobalData (2023), UNICEF (2024), and Macrotrends (2024); ^: sources from World Bank (2025), The Global Economy (2024), SESRIC (2023), and UNICEF (2024); ~: sources from The Global Economy (2024), Statista (2023), and SESRIC (2023); and #: sources from World Health Organization (2024) and UNICEF (2024).

**Table 2:** List of Latent and Manifest Variables with their Respective Loadings by Country.

LV	MV (Unit)	Abbreviation	Loading		
			Bahrain	Kuwait	Qatar
IMR	Infant Mortality Rate, total infant female death (years)	F-IMR	1.000 *	0.991 *	1.000 *
	Infant Mortality Rate, total infant male death (years)	M-IMR	1.000 *	0.991 *	1.000 *
SDOH	Employment to population ratio (females) in a certain year	EMPFEM	0.935 *	0.927 *	-
	Food Production Index in a certain year	FP	0.881 *	0.826 *	0.762 *
	GDP per capita (Local Currency Unit)	GDP	0.974 *	0.858 *	0.907 *
	Gross national income per capita in a certain year	GNI	0.977 *	0.856 *	0.923 *
	Gender of a newborn in a certain year	NEWBORN	0.407 *	0.627 *	-
	Primary School Enrolment in a certain year	PSE	0.963 *	0.690 *	-
	Secondary School Enrolment in a certain year	SSE	-	0.760 *	0.894 *
ENV	CO_2_ emission or oil refineries	CO2EMKT	0.995 *	0.987 *	0.997 *
	Electricity Consumption in a certain year	ECON	0.957 *	0.941 *	0.952 *
	Methane emissions in the energy sector (thousand metric tons of CO_2_ equivalent)	METEMMT	0.994 *	0.941 *	0.990 *
	Methane emissions (kt of CO_2_ equivalent)	METEMKT	0.996 *	0.958 *	0.992 *
	Nitrous oxide emissions (thousand metric tons of CO_2_ equivalent)	NITEM	0.984 *	0.977 *	0.984 *
	Total greenhouse gas emissions (kt of CO_2_ equivalent)	TOTGGEM	0.998 *	0.993 *	0.998 *
HRS	DPT immunization	DPT	0.806 *	0.723 *	0.833 *
	newborns vaccinated with BCG	HBI	0.649 *	0.814 *	0.867 *
	Method needs for family planning with modern contraception methods	MCM	0.954 *	0.853 *	0.933 *
	newborns vaccinated with MCV1 in a certain year	MCV1	0.875 *	0.808 *	0.915 *
	Universal health coverage (UHC) service coverage index in a given year.	UHC	0.857 *	0.799 *	0.781 *

*: Significant at *p* < 0.05. LV: latent variable. MV: manifest variable. SDOH: sociodemographic on health; ENV: environmental factors; HRS: health resources; and IMR: infant mortality rate.

**Table 3:** Reliability, Validity, and Predictability of the Latent Variables by Country.

LV	Country	CA	CR	Rho-A	AVE	Q^2^	R^2^	HTMT (95% CI)		
								HRS	IMR	SDOH
HRS	Bahrain	0.890	0.919	0.927	0.696	0.541	0.823	-	1.004 (0.975, 1.025)	0.926 (0.878, 0.985)
	Kuwait	0.870	0.899	0.895	0.641	0.731	0.752	-	0.944 (0.789, 0.999)	0.922 (0.818, 1.067)
	Qatar	0.918	0.938	0.935	0.753	0.669	0.775	-	0.966 (0.897, 1.003)	0.943 (0.869, 0.999)
SDOH	Bahrain	0.932	0.951	0.974	0.775	0.837	0.845	-	-	-
	Kuwait	0.902	0.924	0.913	0.637	0.822	0.837	-	-	-
	Qatar	0.895	0.928	0.904	0.764	0.889	0.895	-	-	-
ENV	Bahrain	0.995	0.996	0.995	0.974	-	-	0.916 (0.865, 0.969)	0.889 (0.844, 0.927)	0.942 (0.849, 1.015)
	Kuwait	0.986	0.988	0.986	0.934	-	-	0.908 (0.745, 0.996)	0.947 (0.886, 0.996)	0.960 (0.834, 1.016)
	Qatar	0.994	0.995	0.994	0.971	-	-	0.843 (0.709, 0.932)	0.858 (0.783, 0.929)	0.997 (0.931, 1.047)
IMR	Bahrain	1.000	1.000	1.000	1.000	0.764	0.933	-	-	0.883 (0.847, 0.930)
	Kuwait	0.982	0.991	0.983	0.982	0.828	0.883	-	-	0.932 (0.829, 0.986)
	Qatar	1.000	1.000	1.000	1.000	0.709	0.886	-	-	0.969 (0.924, 1.013)

CA: Cronbach’s alpha (>0.7); Rhô -A: rhô-alpha (>0.7); CR: composite reliability (>0.7); AVE: average variance extraction (>0.50); R^2^: >0.25 recommended; Q^2^: 0.35 (strong); SDOH: sociodemographic on health; ENV: environmental factors; HRS: health resources; IMR: infant mortality rate; LV: latent variables; and HTMT: hetero-trait and mono-trait ratio (95% CI < 1.0). All estimates are significant at *p* < 0.05 and 95% CI, 95% confined interval.

**Table 4:** Effect Size, Direct Effect, Indirect Effect, and Total Effect of the Latent Variables by Country.

Hypothesis (Relationship)	Country	Direct Effect (95% CI)	Indirect Effect (95% CI)	Total Effect (95% CI)	f^2^
H1: SDOH → IMR	Bahrain	-	−0.876 (−0.912, −0.850)	−0.876 (−0.912, −0.850)	-
H2: HRS → IMR		−0.966 (−0.987, −0.949)	-	−0.966 (−0.987, −0.949)	13.912
H3: ENV → IMR		-	−0.805 (−0.889, −0.720)	−0.805 (−0.889, −0.720)	-
H4: ENV → SDOH		0.919 (0.829, 0.993)	-	0.919 (0.829, 0.993)	5.442
H5: ENV → HRS		-	0.834 (0.742, 0.921)	0.834 (0.742, 0.921)	-
H6: SDOH → HRS		0.907 (0.873, 0.941)	-	0.907 (0.873, 0.941)	4.654
H1: SDOH → IMR	Kuwait	-	−0.815 (−0.893, −0.791)-	−0.815 (−0.893, −0.791)	-
H2: HRS → IMR		−0.939 (−0.979, −0.909)	-	−0.939 (−0.979, −0.909)	7.518
H3: ENV → IMR		-	−0.745 (−0.845, −0.693)	−0.745 (−0.845, −0.693)	-
H4: ENV → SDOH		0.915 (0.855, 0.971)	-	0.915 (0.855, 0.971)	5.132
H5: ENV → HRS		-	0.794 (0.731, 0.901)	0.794 (0.731, 0.901)	-
H6: SDOH → HRS		0.867 (0.831, 0.944)	-	0.867 (0.831, 0.944)	3.038
H1: SDOH → IMR	Qatar	-	−0.828 (−0.898, −0.788)	−0.828 (−0.898, −0.788)	-
H2: HRS → IMR		−0.941 (−0.976, −0.910)	-	−0.941 (−0.976, −0.910)	7.749
H3: ENV → IMR		-	−0.784 (−0.857, −0.721)	−0.784 (−0.857, −0.721)	-
H4: ENV → SDOH		0.946 (0.887, 0.989)	-	0.946 (0.887, 0.989)	8.527
H5: ENV → HRS		-	0.833 (0.777, 0.897)	0.833 (0.777, 0.897)	-
H6: SDOH → HRS		0.880 (0.847, 0.932)	-	0.880 (0.847, 0.932)	3.440

LV: latent variable; SDOH: sociodemographic on health; ENV: environmental factors; HRS: health resources; IMR: infant mortality rate; and f^2^: effect size 0.35 (strong). All effects are significant at *p* < 0.05 and 95% CI, 95% confident interval.

## Data Availability

Data supporting reported results can be obtained from open sources in the references.

## References

[B1-ejihpe-15-00026] Al-Abdulla S. A., Haj Bakri A., Mansaray M. A., Al-Kuwari M. G. (2024). Assessing the impact of annual health screenings in identifying noncommunicable disease risk factors within Qatar’s primary health care corporation Qatari registered population. Frontiers in Public Health.

[B2-ejihpe-15-00026] Alahmad B., Li J., Achilleos S., Al-Mulla F., Al-Hemoud A., Koutrakis P. (2023). Burden of fine air pollution on mortality in the desert climate of Kuwait. Journal of Exposure Science & Environmental Epidemiology.

[B3-ejihpe-15-00026] Al Boinin H. (2023). Women’s entrepreneurship in the GCC: A literature analysis from a socio-cultural perspective. Journal of Enterprising Communities: People and Places in the Global Economy.

[B4-ejihpe-15-00026] Alenezi G. G., Haridi H. K. (2021). Awareness and use of family planning methods among women in Northern Saudi Arabia. Middle East Fertility Society Journal.

[B5-ejihpe-15-00026] AlRukaibi D., AlSalem A. (2022). A comparative analysis of the carbon dioxide emissions-energy profile in Kuwait: Status quo versus 2030. Journal of Engineering Research.

[B6-ejihpe-15-00026] Alsabah A. M., Alsabti N. H. (2024). Evaluating Public Healthcare Productivity and Health System Efficiency in the State of Kuwait: A Two-Stage Data Envelopment Analysis of Government Hospital Performance. Preprints.

[B7-ejihpe-15-00026] Al-Saidi M., Saliba S. (2019). Water, energy and food supply security in the Gulf Cooperation Council (GCC) countries—A risk perspective. Water.

[B8-ejihpe-15-00026] Anwar A., Ullah I., Younis M., Flahault A. (2021). Impact of air pollution (PM2.5) on child mortality: Evidence from sixteen Asian countries. International Journal of Environmental Research and Public Health.

[B10-ejihpe-15-00026] Braveman P., Egerter S., Williams D. R. (2011). The social determinants of health: Coming of age. Annual Review of Public Health.

[B9-ejihpe-15-00026] Braveman P., Gottlieb L. (2014). The social determinants of health: It’s time to consider the causes of the causes. Public Health Reports.

[B11-ejihpe-15-00026] David J. (2018). Infant mortality and public health expenditure in Nigeria: Empirical explanation of the nexus. Timisoara Journal of Economics and Business (TJE&B).

[B12-ejihpe-15-00026] Dijkstra T. K., Henseler J. (2015). Consistent partial least squares path modeling. MIS Quarterly.

[B13-ejihpe-15-00026] ElMassah S., Hassanein E. A. (2023). Economic development and environmental sustainability in the GCC countries: New insights based on the economic complexity. Sustainability.

[B14-ejihpe-15-00026] Eltayib R. A. A., Al-Alawi K. S., Wirayuda A. A. B., Al-Azri M., Chan M. F. (2023a). The impact of sociodemographic, macroeconomic, and health status and resources determinants on infant mortality rates in the Gulf Cooperation Council (GCC) countries: A systematic review and meta-analysis. Journal of Neonatal Nursing.

[B15-ejihpe-15-00026] Eltayib R. A. A., Al-Azri M., Chan M. F. (2023b). The Impact of Sociodemographic, Macroeconomic, and Health Status and Resources on Infant Mortality Rates in Oman: Evidence from 1980 to 2022. European Journal of Investigation in Health, Psychology and Education.

[B16-ejihpe-15-00026] Fatima T., Elbanna S. (2022). Tackling youth unemployment in GCC region: Reaching beyond national barriers. Academic Network for Development Dialogue (ANDD) Paper Series.

[B17-ejihpe-15-00026] Faul F., Erdfelder E., Lang A.-G., Buchner A. (2007). G* Power 3: A flexible statistical power analysis program for the social, behavioral, and biomedical sciences. Behavior Research Methods.

[B18-ejihpe-15-00026] Gkontelos A., Vaiopoulou J., Stamovlasis D. (2023). Teachers’ innovative work behavior as a function of self-efficacy, burnout, and irrational beliefs: A structural equation model. European Journal of Investigation in Health, Psychology and Education.

[B19-ejihpe-15-00026] GlobalData (2023). Data and insights.

[B20-ejihpe-15-00026] Hair J. F., Risher J. J., Sarstedt M., Ringle C. M. (2019). When to use and how to report the results of PLS-SEM. European Business Review.

[B21-ejihpe-15-00026] Ishimwe M. C. S., Kiplagat J., Knowlton A. K., Livinski A. A., Kupfer L. E. (2023). Reversing the trend: A scoping review of health innovation transfer or exchange from low-and middle-income countries to high-income countries. BMJ Global Health.

[B22-ejihpe-15-00026] Johnson J., Mohamed H., Lowe T., Khraim F., Wolsey C., Haque S., Al-Farsi A., Schnurman D., Chowdhury N., Raihan M. M. H., Turin T. C. (2024). Addressing the effectiveness of health literacy programs within the Gulf Corporation Council: An integrative review. Health Promotion International.

[B23-ejihpe-15-00026] Katoue M. G., Cerda A. A., García L. Y., Jakovljevic M. (2022). Healthcare system development in the Middle East and North Africa region: Challenges, endeavors and prospective opportunities. Frontiers in Public Health.

[B24-ejihpe-15-00026] Kaufmann L., Gaeckler J. (2015). A structured review of partial least squares in supply chain management research. Journal of Purchasing and Supply Management.

[B25-ejihpe-15-00026] Khan M. N., Aziz G., Khan M. S. (2022). The impact of sustainable growth and sustainable environment on public health: A study of GCC countries. Frontiers in Public Health.

[B26-ejihpe-15-00026] Kislyak S., Dugan O., Yalovenko O. (2024). Systems for genetic assessment of the impact of environmental factors. Innovative Biosystems and Bioengineering.

[B27-ejihpe-15-00026] Kreutz J., Heitmann J., Schäfer A.-C., Aldudak S., Schieffer B., Schieffer E. (2023). Environmental factors and their impact on the COVID-19 pandemic. Herz.

[B28-ejihpe-15-00026] Kumar V., Cheng S. Y. C. (2023). A comparative literature review of integrated approach in health care in high and low-middle-income countries. Social Development Issues.

[B29-ejihpe-15-00026] Lee H.-Y., Leslie H. H., Oh J., Kim R., Kumar A., Subramanian S., Kruk M. E. (2022). The association between institutional delivery and neonatal mortality based on the quality of maternal and newborn health system in India. Scientific Reports.

[B30-ejihpe-15-00026] Macrotrends (2024). Global metrics.

[B31-ejihpe-15-00026] Mahfouz M. S., Elmahdy M., Ryani M. A., Abdelmola A. O., Kariri S. A. A., Alhazmi H. Y. A., Almalki S. H. M., Adhabi O. M., Hindi S. M. A., Muqri N. M., Towhary B. A. (2023). Contraceptive use and the associated factors among women of reproductive age in Jazan City, Saudi Arabia: A cross-sectional survey. International Journal of Environmental Research and Public Health.

[B32-ejihpe-15-00026] Marmot M. (2005). Social determinants of health inequalities. The Lancet.

[B33-ejihpe-15-00026] Memon M. A., Ramayah T., Cheah J.-H., Ting H., Chuah F., Cham T. H. (2021). PLS-SEM statistical programs: A review. Journal of Applied Structural Equation Modeling.

[B34-ejihpe-15-00026] Mouteyica A. E. N., Ngepah N. (2023). Health outcome convergence in Africa: The roles of immunization and public health spending. Health Economics Review.

[B35-ejihpe-15-00026] Nazarpour S., Poursani A. S., Simbar M., Yarandi R. B. (2023). The relationship between air pollution and infant mortality rate. Iranian Journal of Public Health.

[B36-ejihpe-15-00026] Our World in Data (2024). Per capita CO_2_ emissions. *Global Change Data Lab*.

[B37-ejihpe-15-00026] Preker A. S., Cotlear D., Kwon S., Atun R., Avila C. (2021). Universal health care in middle-income countries: Lessons from four countries. Journal of Global Health.

[B38-ejihpe-15-00026] Qatar Government Communication Office (2024). Qatar national vision 2030.

[B39-ejihpe-15-00026] Rigdon E. E. (2016). Choosing PLS path modeling as analytical method in European management research: A realist perspective. European Management Journal.

[B40-ejihpe-15-00026] Ringle C. M., Wende S., Becker J.-M. (2024). SmartPLS 4. Bönningstedt: SmartPLS.

[B41-ejihpe-15-00026] Roletto A., Zanardo M., Bonfitto G. R., Catania D., Sardanelli F., Zanoni S. (2024). The environmental impact of energy consumption and carbon emissions in radiology departments: A systematic review. European Radiology Experimental.

[B42-ejihpe-15-00026] Rönkkö M., McIntosh C. N., Antonakis J., Edwards J. R. (2016). Partial least squares path modeling: Time for some serious second thoughts. Journal of Operations Management.

[B43-ejihpe-15-00026] Salam A. A., Al-Khraif R. M. (2020). Child mortality transition in the Arabian Gulf: Wealth, health system reforms, and development goals. Frontiers in Public Health.

[B44-ejihpe-15-00026] Sanchez C. A., Rivera-Lozada O., Lozada-Urbano M., Best P. (2023). Infant mortality rates and pneumococcal vaccines: A time-series trend analysis in 194 countries, 1950–2020. BMJ Global Health.

[B45-ejihpe-15-00026] Sarstedt M., Hair J. F., Nitzl C., Ringle C. M., Howard M. C. (2020). Beyond a tandem analysis of SEM and PROCESS: Use of PLS-SEM for mediation analyses!. International Journal of Market Research.

[B46-ejihpe-15-00026] Schwartz A., Franco J., Campbell J. (2023). The Effects of GDP per capita on infant mortality rates. SACAD: John Heinrichs Scholarly and Creative Activity Days.

[B47-ejihpe-15-00026] Sepanlou S. G., Aliabadi H. R., Malekzadeh R., Naghavi M., GBD Child Mortality in Middle East Collaborators (2022). Neonate, infant, and child mortality in North Africa and Middle East by cause: An analysis for the global burden of disease Study 2019. Archives of Iranian Medicine.

[B49-ejihpe-15-00026] Shannak S. d., Contestabile M. (2022). Does Qatar face a trade-off between economic growth and CO_2_ emissions?. Frontiers in Environmental Science.

[B50-ejihpe-15-00026] Sharma S., Bhardwaj A., Arora K., Akhtar F., Mehra S. (2023). Assessing universal maternal health service coverage and their determinants in India: A multicentric cross-sectional study. Journal of Family Medicine and Primary Care.

[B51-ejihpe-15-00026] Shmueli G., Ray S., Estrada J. M. V., Chatla S. B. (2016). The elephant in the room: Predictive performance of PLS models. Journal of Business Research.

[B52-ejihpe-15-00026] Siah A. K., Lee G. H. (2015). Female labour force participation, infant mortality and fertility in Malaysia. Journal of the Asia Pacific Economy.

[B53-ejihpe-15-00026] Simmons R. A., Anthopolos R., O’Meara W. P. (2021). Effect of health systems context on infant and child mortality in sub-Saharan Africa from 1995 to 2015, a longitudinal cohort analysis. Scientific Reports.

[B54-ejihpe-15-00026] Statista (2023). Statistics: Industry overview.

[B55-ejihpe-15-00026] The Global Economy (2024). Indicators: List of available indicators.

[B56-ejihpe-15-00026] The Office of Disease Prevention and Health Promotion (2024). Healthy people 2030, building a healthier future for all.

[B48-ejihpe-15-00026] The Statistical, Economic, and Social Research and Training Centre for Islamic Countries (SESRIC) (2023). OIC statistics database (OICStat).

[B57-ejihpe-15-00026] Touil H., Mounts K., De Jager P. L. (2023). Differential impact of environmental factors on systemic and localized autoimmunity. Frontiers in immunology.

[B58-ejihpe-15-00026] UNICEF (2024). UNICEF data.

[B59-ejihpe-15-00026] WHO Regional Office for the Eastern Mediterranean (2023). Country cooperation strategy for WHO and Kuwait 2023–2027.

[B60-ejihpe-15-00026] Wirayuda A. A. B., Jarallah S., Al-Mahrezi A., Alsamara M., Barkat K., Chan M. F. (2023). Unlocking the secrets of longevity: Exploring the impact of socioeconomic factors and health resources on life expectancy in Oman and Qatar. Inquiry (United States).

[B61-ejihpe-15-00026] World Bank (2024). Gulf economic update, fall 2023: Structural reforms and shifting social norms to increase women’s labor force participation. *World Bank*.

[B62-ejihpe-15-00026] World Bank (2025). World Bank open data.

[B63-ejihpe-15-00026] World Health Organization (2023). Social determinants of health.

[B64-ejihpe-15-00026] World Health Organization (2024). Data at WHO.

[B65-ejihpe-15-00026] Yasmeen K., Yasmin K., Adnan M., Malik M. (2024). GCC transgender labor market outcomes in GCC. Discover Global Society.

[B66-ejihpe-15-00026] Zaidan A. M., Al-Wathinani A. M., Arbaein T. J. (2024). The effects of toxic air pollutants and environmental health on public health in Saudi Arabia. Polish Journal of Environmental Studies.

